# *NSD1* mutations generate a genome-wide DNA methylation signature

**DOI:** 10.1038/ncomms10207

**Published:** 2015-12-22

**Authors:** S. Choufani, C. Cytrynbaum, B. H. Y. Chung, A. L. Turinsky, D. Grafodatskaya, Y. A. Chen, A. S. A. Cohen, L. Dupuis, D. T. Butcher, M. T. Siu, H. M. Luk, I. F. M. Lo, S. T. S. Lam, O. Caluseriu, D. J. Stavropoulos, W. Reardon, R. Mendoza-Londono, M. Brudno, W. T. Gibson, D. Chitayat, R. Weksberg

**Affiliations:** 1 Program in Genetics and Genome Biology, The Hospital for Sick Children, 555 University Avenue, Toronto, Ontario, Canada M5G 1X8; 2 Division of Clinical and Metabolic Genetics, The Hospital for Sick Children, 555 University Avenue, Toronto, Ontario, Canada M5G 1X8; 3 Department of Molecular Genetics, University of Toronto, 27 King's College Circle, Toronto, Ontario, Canada M5S 1A1; 4 Department of Pediatrics and Adolescent Medicine, Li Ka Shing Faculty of Medicine, University of Hong Kong, 6/F, William MW Mong Block, 21 Sassoon Road, Pokfulam, Hong Kong; 5 Centre for Computational Medicine, The Hospital for Sick Children, 555 University Avenue, Toronto, Ontario, Canada M5G 1X8; 6 Institute of Medical Science, School of Graduate Studies, University of Toronto, 2374-1 King's College Circle, Toronto, Ontario, Canada M5S 1A8; 7 Department of Medical Genetics, UBC, Child and Family Research Institute, 950W 28th Avenue, Vancouver, British Columbia V5Z 4H4, USA; 8 Clinical Genetics Service, Department of Health, Cheung Sha Wan Jockey Club Clinic, 1/F—3/F, 2 Kwong Lee Road, Sham Shui Po, Kowloon, Hong Kong; 9 Department of Medical Genetics, University of Alberta, 116 Street and 85 Avenue, Edmonton, Alberta, Canada T6G 2R3; 10 Pediatric Laboratory Medicine, The Hospital for Sick Children, 555 University Avenue, Toronto, Ontario, Canada M5G 1X8; 11 Laboratory Medicine and Pathobiology, University of Toronto, 27 King's College Circle, Toronto, Ontario, Canada M5S 1A1; 12 Our Lady's Hospital for Sick Children, Crumlin D12 N512 Ireland; 13 Department of Pediatrics, University of Toronto, 27 King's College Circle, Toronto, Ontario, Canada M5S 1A1; 14 Department of Computer Science, University of Toronto, 27 King's College Circle, Toronto, Ontario, Canada M5S 1A1; 15 Prenatal Diagnosis and Medical Genetics Program, Mount Sinai Hospital, 600 University Avenue, Toronto, Ontario, Canada M5G 1X5

## Abstract

Sotos syndrome (SS) represents an important human model system for the study of epigenetic regulation; it is an overgrowth/intellectual disability syndrome caused by mutations in a histone methyltransferase, *NSD1*. As layered epigenetic modifications are often interdependent, we propose that pathogenic *NSD1* mutations have a genome-wide impact on the most stable epigenetic mark, DNA methylation (DNAm). By interrogating DNAm in SS patients, we identify a genome-wide, highly significant *NSD1*^+/−^-specific signature that differentiates pathogenic *NSD1* mutations from controls, benign *NSD1* variants and the clinically overlapping Weaver syndrome. Validation studies of independent cohorts of SS and controls assigned 100% of these samples correctly. This highly specific and sensitive *NSD1*^+/−^ signature encompasses genes that function in cellular morphogenesis and neuronal differentiation, reflecting cardinal features of the SS phenotype. The identification of SS-specific genome-wide DNAm alterations will facilitate both the elucidation of the molecular pathophysiology of SS and the development of improved diagnostic testing.

## Abstract (web summary)

Sotos syndrome is an growth syndrome characterized by advanced growth in childhood, characteristic facial appearance and intellectual disability. Here the authors identify a genome-wide DNA methylation signature that accurately diagnoses Sotos Syndrome and distinguishes it from similar conditions.

## Graphical abstract

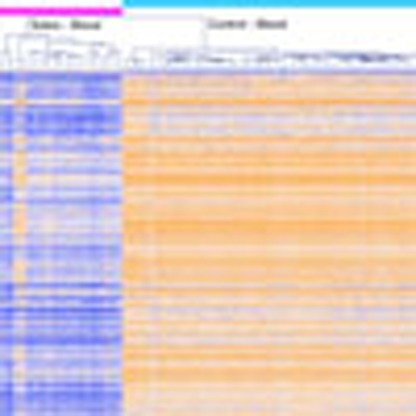

Recent advances in next-generation sequencing technologies have led to the discovery of the molecular basis of many overgrowth syndromes. Constitutional mutations in two different genes involved in regulating histone modifications, *NSD1* and *EZH2*, have been shown to cause clinically overlapping overgrowth disorders, Sotos syndrome (SS) and Weaver syndrome, respectively.

Sotos syndrome (OMIM 117550) is an autosomal dominant condition with an estimated prevalence of 1:14,000 live births (Rahman, unpublished data). It is characterized by pre- and postnatal overgrowth, advanced bone age, distinctive facial gestalt and a variety of neurodevelopmental problems including intellectual disability^1^. Mutations in *NSD1* (nuclear receptor SET (su(var)3–9, enhancer-of-zeste, trithorax) domain containing protein-1) are found in 80–90% of patients with SS^2^,^3^,^4^. As mutations in other genes have not been reported in SS patients, the remaining 10–20% likely harbour undetected *NSD1* mutations or represent clinical misclassification.

*NSD1* is one of many genes that have been recently recognized to developmentally regulate the epigenome. *NSD1* encodes a histone H3 lysine 36 methyltransferase, important for multiple aspects of normal embryonic development^5^. NSD1 binds near various promoter elements to regulate transcription via interactions with H3K36 methylation and RNA polymerase II (ref. 6). In general, it has been proposed that during development, histone modifications are fairly transient regulatory marks that are replaced in the longer term by the more stable epigenetic mark DNAm^7^. As a proof of principle of the interaction between loss of function in a histone modifier and DNAm status, we have previously demonstrated that mutations in a lysine-specific demethylase, *KDM5C,* generates specific genome-wide DNAm alterations^8^. Here we analyse the DNA methylome in SS patients and define a specific genome-wide pattern of DNAm alterations associated with *NSD1* loss-of-function mutations. We demonstrate how this profile may be used to derive a molecular *NSD1*^+/−^-specific signature with high sensitivity and specificity and validate this signature in independent cohorts of SS and controls. We show that the signature is able to distinguish pathogenic *NSD1* mutations not only from control subjects, but also from benign sequence variants within *NSD1* and from cases with the clinically overlapping Weaver syndrome caused by mutations in the histone methyltransferase *EZH2* (Enhancer of Zeste, Drosophila, Homolog 2). Finally, we determine the functional significance of the observed genome-wide DNAm signature and demonstrate its potential utility in the diagnostic testing of *NSD1* mutations of unknown significance.

## Results

### Identification of *NSD1* ^+/−^-specific DNAm signature

To determine whether *NSD1* loss-of-function mutations impact stable epigenetic marks such as DNAm at downstream genomic targets, we compared DNAm in peripheral blood from SS patients with known pathogenic *NSD1* mutations (*NSD1*^*+/*−^*; n*=19) to controls (*n*=53) (Supplementary Data 1 and 2). The demographics for the discovery cohort were as follows: For SS, there were 11 males and eight females and the mean age±s.d. at sample collection was 10.1±9.6 years (range 0.6–40 years). The 53 control subjects included 24 males and 29 females; their mean age at the time of sample collection was 10.8±4.8 years (range 1–21 years). There was no statistically significant difference between SS and controls with regard to the age at which blood was sampled (Wilcoxon rank-sum test, *P* value=0.216) or sex (Chi-square (*χ*2) test, *P* value=0.345).

Genomic DNA was treated with sodium bisulfite and DNAm was assessed using the Illumina Human InfiniumMethylation450 BeadChip. After filtering for polymorphic single-nucleotide polymorphisms (SNPs) and nonspecific probes as previously described^9^, we quantified DNAm at 424,586 CpG sites using beta scores, which represent DNAm levels as a percentage (between 0 and 1). The significance of differential DNAm between SS and control samples was assessed at each CpG using a non-parametric Mann–Whitney *U*-test with a stringent Bonferroni correction for multiple testing.

**Figure 1:**
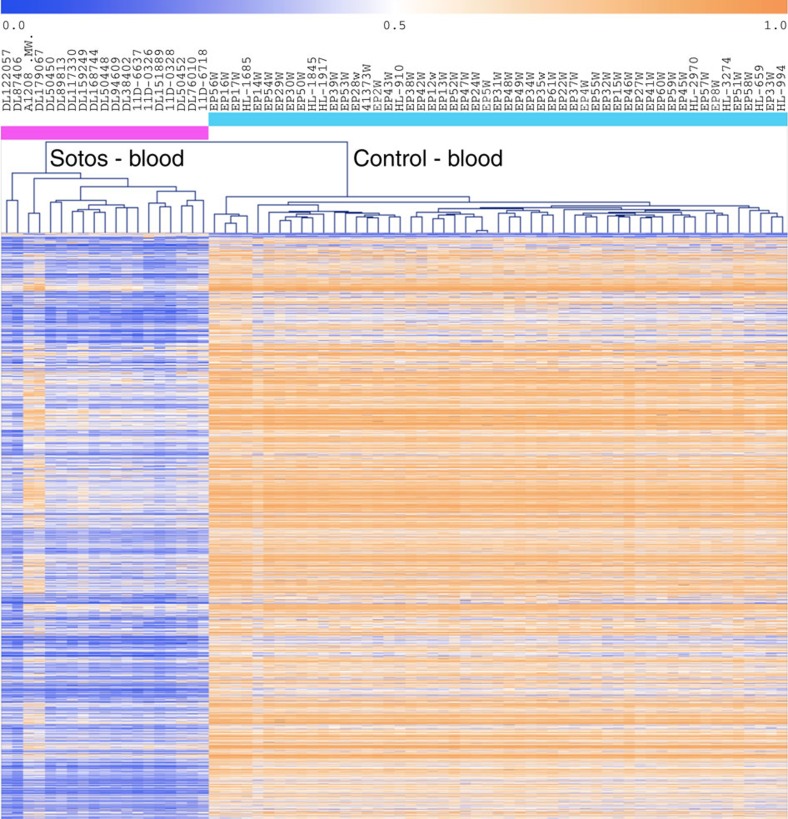
DNA methylation signature associated with *NSD1*^*+/*−^ mutations. Unsupervised hierarchical clustering of 72 samples using the differentially methylated CpG sites comprising the *NSD1*^+/−^-specific signature is shown using Pearson correlation. Note that two patients (A1208 and DL179067) harbour the same mutation in *NSD1* at the end of the gene (exon 22) and display slightly different DNA methylation changes compared with the other SS patients. Orange indicates high DNA methylation and blue indicates low DNA methylation. Pink bar represents SS patients with *NSD1* whole-gene deletion and truncating mutations. Blue bar represents controls.

We identified genome-wide changes in DNAm in SS compared with controls. Over 28,000 CpG sites survived stringent Bonferroni correction at *q*<0.05 (Supplementary Fig. 1) demonstrating a previously unrecognized effect of *NSD1*^*+/*−^ on genome-wide DNAm. Next, we applied an additional filter to this highly statistically significant set of CpGs by selecting the subset of probes with >20% difference in average DNAm levels between SS and controls (Supplementary Fig. 1). We identified 7,085 CpG sites distributed across the genome that we refer to as the *NSD1*^+/−^-specific signature; 7,038 CpG sites (99.3%) demonstrated loss of DNAm (Supplementary Data 3), whereas only 47 CpG sites (0.7%) showed a gain of DNAm in SS compared with controls. Using unsupervised hierarchical clustering of the DNAm data for the *NSD1*^+/−^-specific signature, all *NSD1*^+/−^ samples clustered as a distinct group separate from controls (Fig. 1). When tested against several potential confounding factors such as sex, age, batch (Supplementary Data 3) and cell-type composition (Supplementary Fig. 2, see Supplementary Methods for details). the *NSD1*^+/−^-specific signature retained its ability to discriminate SS from control samples.

### Validation of the *NSD1* ^+/−^-specific signature

**Figure 2:**
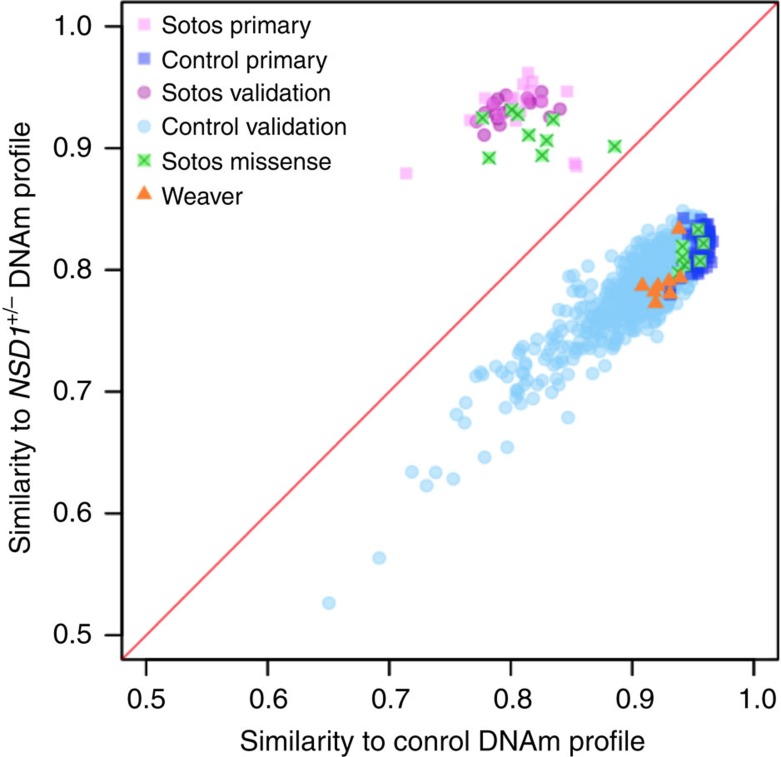
Testing the sensitivity and the specificity of the *NSD1*^+/−^ DNAm signature. Using 19 SS and 53 healthy subjects from the discovery cohort (labelled as ‘primary'), we generated the median-methylation profiles of SS and control, respectively, on the CpG sites comprising the *NSD1*^+/−^-specific signature. We then estimated the specificity of these profiles on a validation set of GEO blood samples (*n*=1,056, blue dots), all of which were more similar to the control profile (specificity 100%). We also estimated the sensitivity on a separate SS validation set (*n*=19, magenta dots), all of which were more similar to the SS profile (100% sensitivity). Similarity was computed as the Pearson correlation to either the SS or the control DNAm profile. Out of 16 missense samples (green squares), 9 classify with SS and 7 with controls. Also shown are Weaver patients with *EZH2* mutations (orange triangles) as well as the classification of the primary 53 controls (dark blue squares) and 19 SS (light magenta squares) used in the derivation of the *NSD1*^*+/*−^-specific signature.

Using the DNAm beta values at each CpG within the *NSD1*^+/−^-specific signature, we developed a predictive model that classifies new subjects on the basis of their DNAm profile as either ‘SS' or ‘not SS', using the SS score described in the Methods. We evaluated the performance of the *NSD1*^+/−^-specific signature using an independent set of normal blood-derived DNAm data (*n*=1,056 subjects) extracted from the Gene Expression Omnibus (GEO) database (www.ncbi.nlm.nih.gov/geo/) (Supplementary Data 4). Each of the 1,056 GEO samples received a negative SS score and was classified as ‘not SS' (Fig. 2), demonstrating 100% specificity of the classification model. These results highlight the robustness of the *NSD1*^+/−^-specific signature as it overcame many sources of variation (sex, age, batch, DNA isolation methods, cell-type composition) introduced by processing different cohorts in different laboratories around the world.

We then tested the sensitivity of the SS score to predict the pathogenicity of *NSD1* mutations using a replication cohort of SS cases with known pathogenic *NSD1* mutations (*n*=19) from Hong Kong (Supplementary Data 5). Each of these patients received a positive SS score (Fig. 2) demonstrating a sensitivity of 100%.

**Figure 3:**
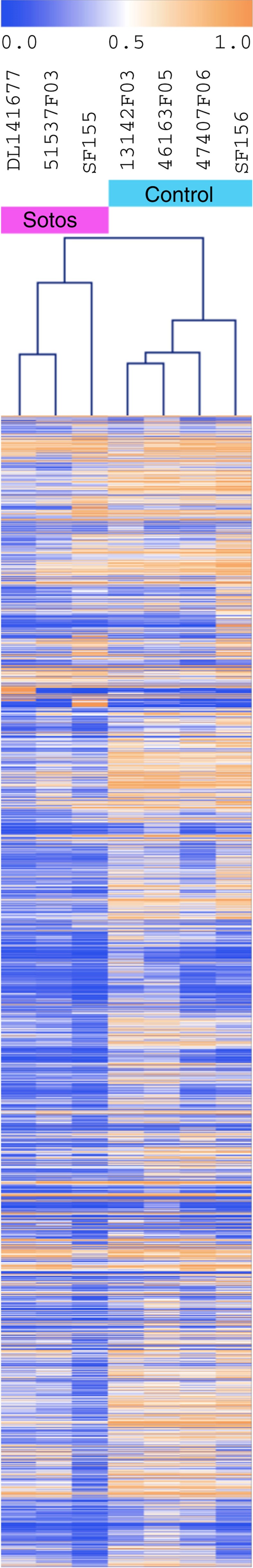
*NSD1*^*+/*−^-specific signature can be identified in SS fibroblasts. Unsupervised hierarchical clustering of three SS-derived fibroblasts (Supplementary Data 1) and four control-derived fibroblasts clearly distinguish SS from controls. Orange indicates high DNA methylation and blue indicates low DNA methylation. Pink bar represents Sotos samples with *NSD1* whole-gene deletion and truncating mutations. Blue bar represents controls.

As DNAm can be tissue and cell-type specific, we tested fibroblast-derived DNA from three SS patients with truncating mutations in *NSD1* in comparison to four control fibroblast samples. Using hierarchical clustering and the *NSD1*^*+/*−^-specific signature derived from blood to assess the fibroblast DNAm data, the three SS fibroblast samples could be clearly distinguished from controls (Fig. 3). Despite the small size of the available fibroblast data set, these results demonstrate that the *NSD1*^+/−^-specific signature is robust even in the context of cell-type-specific DNAm profiles.

To further assess the specificity of the *NSD1*^*+/*−^-specific signature, we used it to analyse the DNAm profiles of eight patients with a clinical diagnosis of Weaver syndrome (OMIM 277590) and confirmed mutations in *EZH2* (refs 10, 11; Supplementary Data 6). All Weaver syndrome patients with *EZH2*^+/−^ mutations received a strongly negative SS score (between −0.151 and −0.105) and were therefore classified confidently as ‘not SS' (Fig. 2). The fact that the *NSD1*^+/−^-specific signature allows the molecular distinction of two clinically overlapping overgrowth syndromes provides further evidence for the robust specificity of the *NSD1*^*+/*−^*-*specific signature.

### Mutation variants of unknown significance

The interpretation of non-synonymous variants (variants of unknown significance or VOUS) represents a significant challenge in the clinical setting. Several different pathogenicity prediction algorithms have been developed to assist in the interpretation of VOUS; however these tools often provide incongruent results^12^.

To investigate the utility of the *NSD1*^+/−^-specific signature to functionally classify *NSD1* VOUS, DNA samples from 16 individuals with missense mutations in *NSD1* (6 from the discovery cohort and 10 from the validation cohort) were tested in a blinded fashion. As shown in Fig. 2, the *NSD1*^+/−^-specific signature allowed clear classification of VOUS as pathogenic or benign; 9/16 samples received positive SS scores clustering with the *NSD1*^+/−^ pathogenic variants; these mutations were classified as pathogenic. The remaining seven samples received negative SS scores, clustering with the control group; these mutations were classified as benign variants.

**Figure 4:**
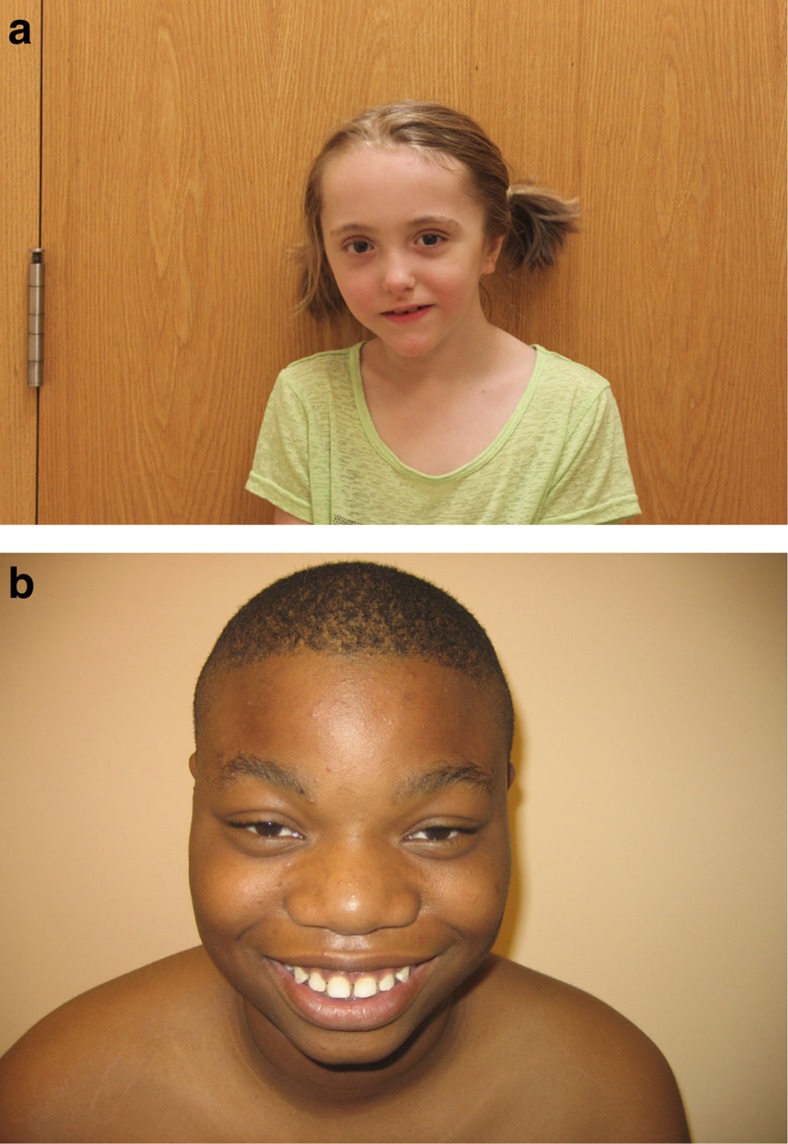
Photographs of two patients carrying *NSD1* missense variants. (**a**) Patient (DL136303) with a missense mutation of uncertain significance, clinical presentation characteristic of Sotos syndrome and a positive SS score based on our analysis. (**b**) Patient (DL181344) with a missense mutation of uncertain significance, clinical presentation that is not characteristic of Sotos syndrome and a negative SS score based on our analysis. Specific consent to publish facial photographs was obtained for the two patients.

To further evaluate the efficacy of the DNAm signature as a tool to classify *NSD1* VOUS as benign or pathogenic, we compared the congruence of results from DNAm SS scores with expert review of the clinical phenotype. Two highly experienced clinical geneticists (RW and DC), who were blinded to the molecular data, reviewed clinical information and photos. Patients were classified into one of three phenotypic categories: (1) typical SS, (2) possible SS and (3) unlikely SS. For the discovery cohort, there was 100% concordance (6/6) for the clinical classification between reviewers. Four patients were categorized as typical SS, all of whom had the DNAm signature. Two patients were categorized as unlikely SS, neither of whom had the signature (Fig. 4, Table 1 and Supplementary Data 7). For the validation cohort, only 5 out of 10 patients had photographs and medical records available that met our criteria for review; the clinicians' assessments were again 100% concordant. Two of the patients were categorized by both clinicians as unlikely SS; these patients did not have the methylation signature. Two of the patients were categorized by both clinicians as typical SS; these patients did have the methylation signature. One patient with the methylation signature was categorized by both clinicians as possible SS.

We also compared the interpretation of *NSD1* VOUS (16 missense variants) using the DNAm signature versus five independent prediction algorithms, namely PolyPhen-2 (ref. 13; http://genetics.bwh.harvard.edu/pph2/), Mutation Assessor (http://mutationassessor.org)^14^, SIFT (http://sift-dna.org)^15^, Mutation Taster (http://www.mutationtaster.org)^16^ and PMut (http://mmb.irbbarcelona.org/PMut/)^17^. As shown in Table 1, the presence of the *NSD1*^+/−^-specific signature and positive SS scores were in agreement with the pathogenicity of the variants predicted by four out of five prediction algorithms. In contrast, negative SS scores were only in agreement with PolyPhen-2 and Mutation Assessor. Taken together, these data demonstrate that prediction of pathogenicity using the *NSD1*^+/−^-specific signature is more congruent with diagnostic classification by expert clinicians in comparison with the majority of the currently available prediction algorithms.

We propose that our specific genome-wide DNAm signature for pathogenic *NSD1* mutations can be utilized as a novel epigenomic diagnostic tool that will facilitate the classification of VOUS in *NSD1* as benign or pathogenic variants.

### Functional significance of the SS classification signature

**Figure 5:**
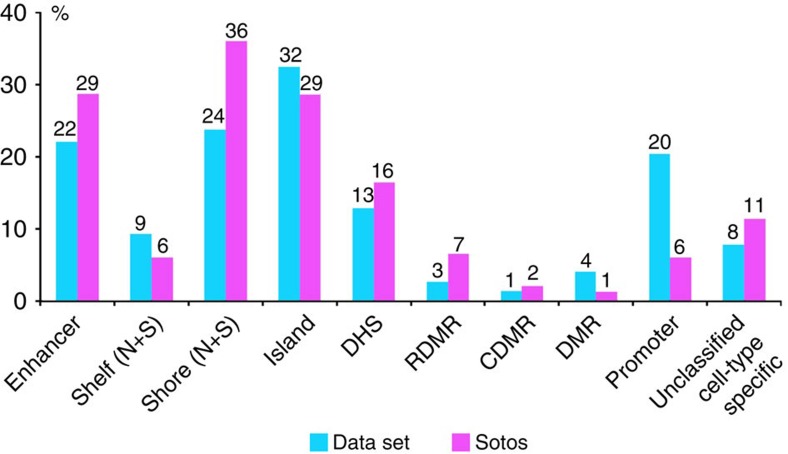
Genomic distribution of the *NSD1*^+/−^-specific CpG sites. Bar chart representing the percentage distribution of the CpG sites according to genomic annotations extracted from the Illumina 450K array annotation file. We compared the distribution of the CpGs between the data set (424,586 CpGs) and *NSD1*^+/−^ CpGs (7,085 CpGs) for the regulatory feature group, relation to CpG island and other functional categories such as overlapping enhancer region, DNase hypersensitive sites (DHS) and type of differentially methylated regions (Reprogrammed DMR (RDMR), cancer DMR (cDMR), other DMR) in addition to relation to RefSeq group annotation. The numbers at the top of each bar represent the percentage distribution of CpGs within each category. N refers to north and S refers to south.

Finally, we investigated the DNAm classification signature of *NSD1*^+/−^ for its potential to elucidate the molecular pathophysiology of SS. Analysis of the genomic locations of the CpG sites in the *NSD1*-specific signature showed that CpGs were over-represented in enhancers, DNase hypersensitive sites, reprogrammed differentially methylated regions (RDMR) and CpG island shores (defined as 0 to 2 kb upstream of CpG island), and were under-represented in regions overlapping promoters and CpG islands (Fig. 5 and Supplementary Data 10). These findings correlate with one previous observation that NSD1 associates primarily within a region ∼1,200 bp upstream of gene targets such as the bone-morphogenetic protein *BMP4* start site^6^. However, our data suggest that NSD1 may have multiple functions depending on the genomic locus and the temporal/spatial location in development—specifically that NSD1 binds regulatory regions that are required for early embryonic development as also reflected by the embryonic lethality at E10.5 of the *Nsd1* null mouse^18^.

**Figure 6:**
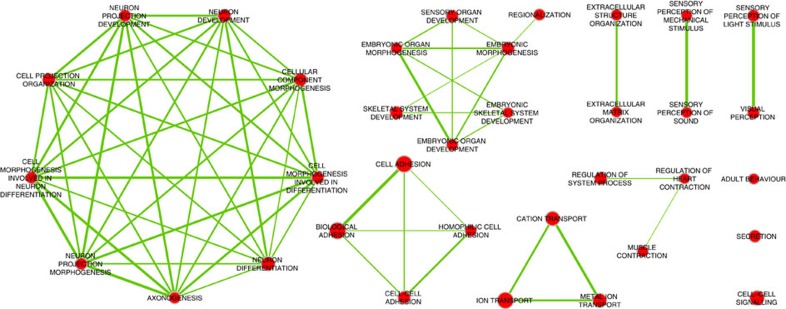
Enrichment analysis of the genes overlapping *NSD1*^+/−^ DNAm signature. We identified 2,167 unique genes that overlap the CpGs comprising the *NSD1*^+/−^-specific signature and used DAVID (http://david.abcc.ncifcrf.gov) to identify the biological processes most enriched within our data set. Over-represented functional categories were visualized in Cytoscape (http://www.cytoscape.org) using the Enrichment Map plugin (www.baderlab.org/Software/EnrichmentMap). Network nodes represent statistically significant Gene Ontology—Biological Process terms (Benjamini-corrected *P* value <0.05), with node size proportional to the number of SS-associated genes annotated for each term. Edges represent overlaps between these gene sets, with edge thickness proportional to their Jaccard index. The results demonstrate enrichment in functional terms that relate to cellular morphogenesis, neuronal development and cellular differentiation, involving highly overlapping subsets of genes. In addition, we identified an enrichment of genes with roles in organ development, ion transport as well as embryonic developmental pathways.

To identify the biological processes and molecular functions most enriched within our data set, we analysed the 2,167 unique genes that overlapped the *NSD1*^+/−^-specific signature using DAVID (http://david.abcc.ncifcrf.gov)^19^. The results demonstrate enrichment for genes with roles in cellular morphogenesis and differentiation, as well as neuronal differentiation/axonogenesis and cell adhesion/cell signalling (Benjamini–Hochberg corrected *q*<0.05) (Fig. 6 and Supplementary Data 8). This enrichment in neural and cellular development pathways reflects the cardinal features of SS (that is, overgrowth and developmental delay) and validates the utility of the DNAm signature to elucidate the functional, biological and molecular impact of *NSD1* pathogenic variants.

We also used GREAT (http://great.stanford.edu)^20^ to directly analyse the 7,085 CpGs within the classification signature, comparing them to the initial 424,586 CpGs in our data set in the context of broader functionally annotated regions (up to 1 Mbp extension from the nearest genes). Functional categories related to embryonic development, including neurodevelopment, were again predominant among the detected enrichment patterns (Supplementary Data 9).

## Discussion

We have identified a novel, robust DNAm signature specifically associated with pathogenic *NSD1* gene mutations, which has the potential to be utilized as a functional molecular test to assess VOUS in *NSD1.* This study will set the stage for an unprecedented new field of epigenetic diagnostic testing where scientists and clinicians will harness the power of the methylome to unravel the pathogenicity of VOUS. We expect that this approach will be particularly valuable with regard to genetic testing for a variety of overgrowth disorders particularly as mutations in additional epigenetic regulators, histone-lysine *N*-methyltransferase (*SETD2)* (ref. 21) and DNA methyltransferase 3 (*DNMT3A)*^22^, have recently been reported to cause distinct overgrowth syndromes.

Our data strongly suggest that there is important crosstalk between histone modifications and DNAm. Although there are currently limited data regarding the mechanisms by which this occurs, one study of *NSD1* loss of function supports the concept of crosstalk between DNAm, histone modifications and gene expression. Specifically, Berdasco *et al.*^23^ report changes in histone modifications and transcription at one genomic locus in tumours following epigenetic silencing of *NSD1* as well as one lymphoblast cell line from a SS individual^23^. This study did not, however, examine genome-wide DNAm. To pursue our interest in the mechanisms by which germline *NSD1* loss-of-function mutations impact the DNA methylome, we are currently establishing a developmental model system to study *NSD1* in induced pluripotent stem (iPS) cells. We anticipate that these studies will further elucidate the mechanisms by which interactions between histone modifications and DNAm drive gene expression.

Finally, the genome-wide DNAm alterations identified by the *NSD1*^+/−^-specific signature represent novel, powerful and highly specific targets that can be used to elucidate the molecular pathophysiology of SS and to guide the development of future targeted therapies. This approach could be used in a broader context to study the downstream functional effects of loss-of-function mutations of regulators of the epigenome in human disease model systems.

## Methods

### DNA methylation analyses

We profiled a total of 112 samples from 114 unique individuals (57 with *NSD1* mutations or single-nucleotide variants and 57 controls). Informed consent was obtained from parents of all the participants and assent was obtained from participants, as appropriate for age. The study was approved by the Research Ethics Board at the Hospital for Sick Children. Most DNA samples were derived from blood except for seven samples (three with *NSD1* mutations and four controls) for which DNA was derived from skin fibroblasts. DNA samples were sodium bisulfite converted using the Qiagen EZ DNA Methylation kit (Qiagen, Valencia, CA), according to the manufacturer's protocol. Modified genomic DNA was then processed and analysed on the Infinium HumanMethylation450 BeadChip from Illumina (Illumina 450K) according to the manufacturer's protocol. The distribution of the samples on the arrays was randomized for both cases and controls but not for age and gender. Chi-square and Wilcoxon rank-sum tests were used to compare gender and age distributions, respectively between SS and control discovery cohorts.

### Normalization and quality controls

We used the GenomeStudio software from Illumina to process the raw intensity data (IDAT files) for all the 114 samples. Control normalization and background subtraction included in GenomeStudio was used to generate DNAm profiles, or beta values, for each sample at every CpG site from the ∼485,000 CpG sites. All the 114 samples passed the quality controls measures and had over 485,000 CpG sites detected at a detection *P* value <0.01.

### Probe cross reactivity and polymorphic sites

We excluded probes containing SNPs, that is, whenever the CpG sites were polymorphic at the cytosine or the guanine base. Infinium I probes were excluded if there was a SNP at the position where single-base extension occurs. Also excluded were CpG sites for which SNPs were located within 10 bases of the query site where single-base extension occurs. SNPs were also excluded if there was an allele frequency of at least 1% (19,418 sites (4.0%)) or an allele frequency of at least 5% (10,825 sites (2.2%); for more details, see Chen *et al.*)^9^. In addition, we excluded CpG sites if their probe sequences aligned to multiple positions with ≥90% identity (see Chen *et al.*,^9^ for additional details). After removing probes with missing values or detection *P* values >0.01 and nonspecific probes, the final data set contained 424,586 probes.

### Differential DNA methylation analysis

To identify the differentially methylated CpG sites, we compared the DNAm distributions for Sotos cases versus controls at each CpG site. To account for the influence of the family relationships among three of the SS patients, we formed three separate testing trials, each time combining 16 non-familial Sotos cases with only one family member. The resulting set of 17 SS patients was compared with 53 controls for each of the 424,586 available CpG sites, using a non-parametric Mann–Whitney *U*-test (implemented in R, scripts available upon request). A stringent Bonferroni correction for multiple testing was applied to the results in each trial. To ensure robust results, we retained only the CpG sites that were significant at the confidence level *α*=0.05 in all the three trials, that is, with any choice of the family representative among the Sotos patients. As many as 28,458 CpG sites satisfied this criterion. Finally, we applied an additional effect-size criterion requiring at least 20% difference in average DNAm between the Sotos and the control groups in each of the three trials. The latter filter was performed to ensure inclusion of CpGs with differences that were the most biologically meaningful. This filter reduced the *NSD1* signature to 7,085 CpG sites, which were then selected for further characterization including the development of an independent classification model for cohorts of controls and SS cases, as well as for specificity and sensitivity testing.

### Sotos syndrome score and classification model

We developed a simple classification model on the basis of the *NSD1*^+/−^-specific signature in blood. At each of the 7,085 *NSD1*^+/−^-specific signature CpGs, a median DNAm level was computed across all the 19 SS patients in the original Discovery cohort. This resulted in a reference profile for the *NSD1*^*+/*−^ Sotos DNAm levels over the *NSD1*^+/−^-specific signature CpGs, which was robust to outliers. Similarly a robust median-DNAm reference profile for the 53 healthy control subjects was created. The classification of each new DNAm sample was based on extracting a vector *B*_sig_ of its DNAm values in the *NSD1*^+/−^-specific signature CpGs, and comparing *B*_sig_ to the two reference profiles computed above. A Sotos Syndrome Score was defined as:


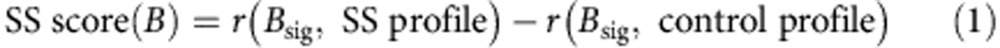


where *r* is the Pearson correlation coefficient. A simple classification model was developed based on scoring each new DNAm sample using the SS score: a blood sample with a positive SS score is more similar to the SS reference profile based on the *NSD1*^+/−^-specific signature CpGs, and is therefore classified as “SS”; whereas a sample with a negative SS score is more similar to the normal-blood reference profile, and is classified as “not SS”. The classification is implemented in R (scripts available upon request).

## Additional information

**Accession codes:** The DNA methylation data were deposited in GEO database under the accession number GSE74432.

**How to cite this article:** Choufani, S. *et al.* *NSD1* mutations generate a genome-wide DNA methylation signature. *Nat. Commun.* 6:10207 doi: 10.1038/ncomms10207 (2015).

## Supplementary Material

Supplementary Information Supplementary Figures 1-2, Supplementary Methods and Supplementary References

Supplementary Data 1 Sotos patients with NSD1 loss-of-function mutations

Supplementary Data 2 Sample Information-Control Discovery Cohort

Supplementary Data 3 List of NSD1+/- significant CpG sites.

Supplementary Data 4 List of samples extracted from GEO for specificity analysis

Supplementary Data 5 Sotos patients with NSD1 (NM_022455.4,NCBI hg18) loss of function mutations-Replication study

Supplementary Data 6 Weaver patients with EZH2 (NM_004456.4,NCBI hg19) mutations/deletions

Supplementary Data 7 Overgrowth patients with NSD1 (NM_022455.4,NCBI hg18) missense variants

Supplementary Data 8 DAVID enrichment in GO Biological Process - FAT ontology for classification signature genes.

Supplementary Data 9 GREAT enrichment in GO Biological Process ontology for classification signature CpG set (hypergeometric test).

Supplementary Data 10 Distribution of the NSD1 specific DNA methylation signature based on annotated genomic features

## Figures and Tables

**Table 1:** Comparison between different prediction algorithms and *NSD1* ^*+/−*^-specific signature for missense mutations.

**Sample ID**	**Protein change**	**Inheritance**	***NSD1*** **DNAm signature**	**Sotos syndrome score**	**Clinical impression (RW and DC)**	**PolyPhen-2 prediction effect (score)**	**SIFT (score)**	**Mutation assessor: functional impact (score)**	**PMut prediction (reliability)**	**Mutation taster (*****P***-**value)**
HK-5474	p.Cys1606Tyr	*De novo*	Yes	0.096	Photos not available	Probably damaging (1)	Deleterious (0)	Medium (3.37)	Pathological (9)	Disease causing (1)
HK-11693	p.Pro1726Arg	*De novo*	Yes	0.122	Photos not available*	Probably damaging (1)	Deleterious (0)	High (3.61)	Pathological (7)	Disease causing (1)
HK-5581	p.Val1968Ala	*De novo*	Yes	0.016	Typical Sotos	Probably damaging (1)	Deleterious (0)	High (4.11)	Neutral (1)	Disease causing (1)
HK-3326	p.Tyr1997Cys	*De novo*	Yes	0.089	Possible Sotos	Probably damaging (1)	Deleterious (0)	High (4.75)	Pathological (8)	Disease causing (1)
HK-435	p.Arg2017Trp	*De novo*	Yes	0.131	Typical Sotos	Probably damaging (1)	Deleterious (0)	High (4.89)	Pathological (9)	Disease causing (1)
**DL136303**	p.Ala1927Pro	Unknown	Yes	0.110	Typical Sotos	Probably damaging (1)	Deleterious (0)	Medium (3.12)	Pathological (3)	Disease causing (1)
**DL208122**	p.Cys2146Ser	*De novo*	Yes	0.077	Typical Sotos	Probably damaging (1)	Deleterious (0)	High (3.89)	Pathological (1)	Disease causing (1)
**DL199861**	p.Cys2138Arg	*De novo*	Yes	0.068	Typical Sotos	Probably damaging (1)	Deleterious (0)	High (3.89)	Pathological (8)	Disease causing (1)
**DL159025**	p.Arg2005Gly	*De novo*	Yes	0.148	Typical Sotos	Probably damaging (1)	Deleterious (0)	Medium (2.35)	Pathological (6)	Disease causing (1)
**DL181344**	p.Asn1650Ser	Unknown	No	−0.141	Unlikely Sotos	Benign (0)	Tolerated (0.09)	Low (1.08)	Pathological (0)	Disease causing (0.9)
HK-9776	p.Asn357Ser	Mat. inheritance	No	−0.122	Unlikely Sotos	Benign (0.1)	Tolerated (0.73)	Neutral (−0.81)	Neutral (5)	Polymorphism (0.9)
HK-12366	p.Asn1149Ser	Mat. inheritance	No	−0.131	Photos not available	Benign (0)	Tolerated (0.28)	Neutral (0.20)	Neutral (2)	Polymorphism (1)
HK-6943	p.Pro2225Gln	Mat. inheritance	No	−0.139	Photos not available	Benign (0)	Tolerated (0.14)	Neutral (0.55)	Neutral (0)	Polymorphism (0.9)
HK-14867	p.Gln2474Arg	*De novo*†	No	−0.121	Unlikely Sotos	Benign (0)	Deleterious (0.01)	Neutral (0.34)	Neutral (1)	Polymorphism (1)
HK-11767	p.Lys1786Arg	Mat. inheritance	No	−0.149	Photos not available	Benign (0.2)	Deleterious (0)	Neutral (0.36)	Neutral (8)	Disease causing (0.9)
**DL73286**	p.Ser1241Thr	Mat. inheritance	No	−0.137	Unlikely Sotos	Benign (0)	Tolerated (0.14)	Neutral (0.55)	Neutral (8)	Polymorphism (1)

Sample IDs representing the discovery cohort are in bold and the remaining samples are from the validation cohort.

Single-nucleotide variants were classified on the basis of DNA methylation data, clinical assessment and mutation databases. Each variant was classified by each of the mutation effect prediction algorithms independently.

^*^Photos available, but did not meet inclusion criteria for clinical review.

^†^Both parents tested negative for the variant in *NSD1*. Also, non-paternity was ruled out in this *de novo* variant case.
