# Supplementary material for: NSD1 mutations generate a genome-wide DNA methylation signature
Source: Nat Commun. 2015 Dec 22;6:10207. doi: 10.1038/ncomms10207 (PMC4703864; doi:10.1038/ncomms10207)
Supplement: Supplementary Information — Supplementary Figures 1-2, Supplementary Methods and Supplementary References [file ncomms10207-s1.pdf]

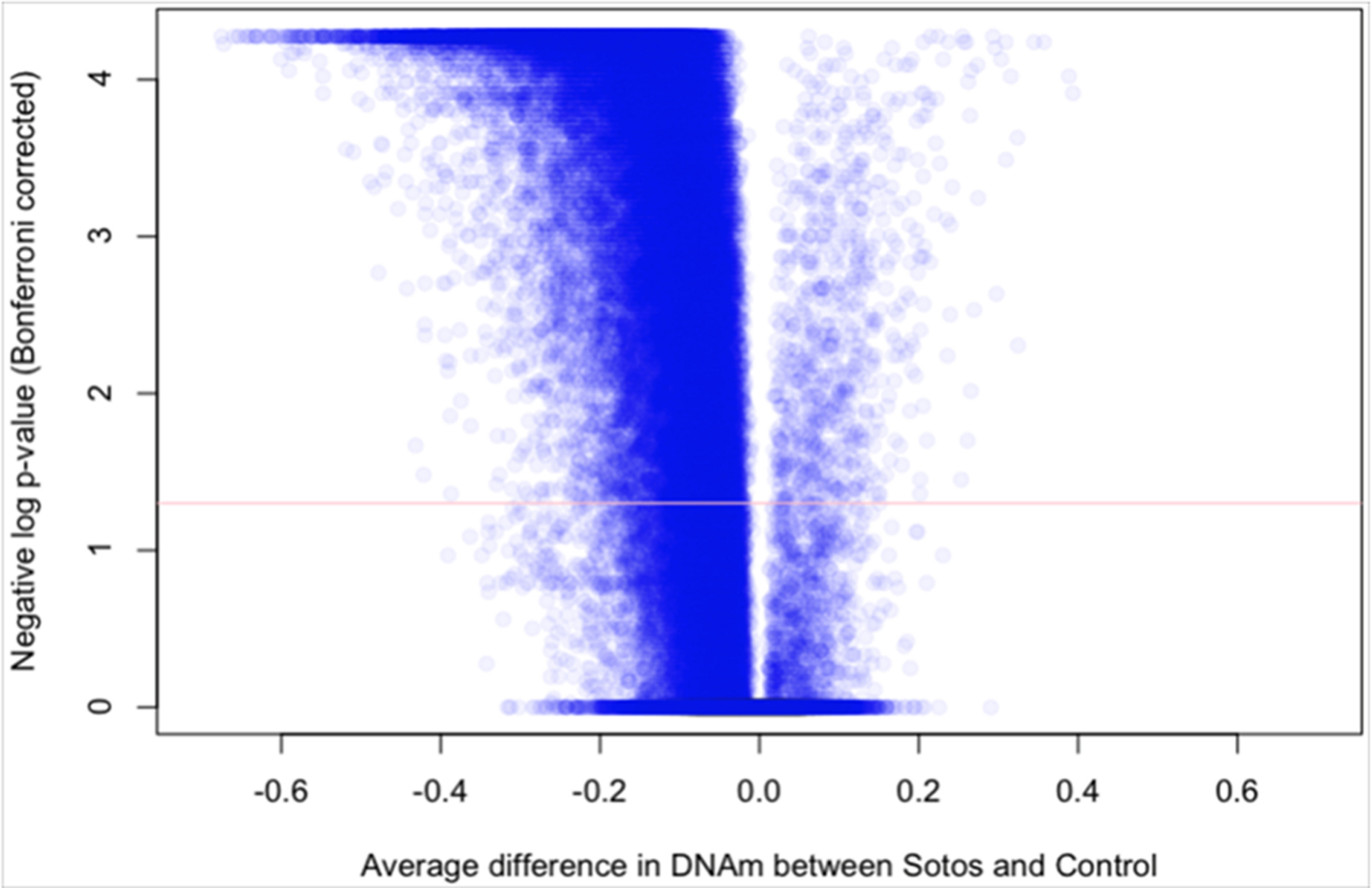

Supplementary Figure 1. Identification of differentially methylated CpGs between SS and control subjects. The volcano plot displays the relationship between the difference in DNAm levels and the significance between the two groups, using a scatter plot. The y-axis is the negative log10 of p-values after Bonferroni correction (a higher value indicates greater significance) and the x-axis is the difference in average DNAm between SS and control subjects. The red horizontal line depicts the significance level at p<0.05. Note that most of the significant CpGs have lower DNAm levels in SS compared to control. At a 20% cut-off for average DNAm differences, we were able to clearly enrich for highly significant CpGs that distinguish SS from controls.

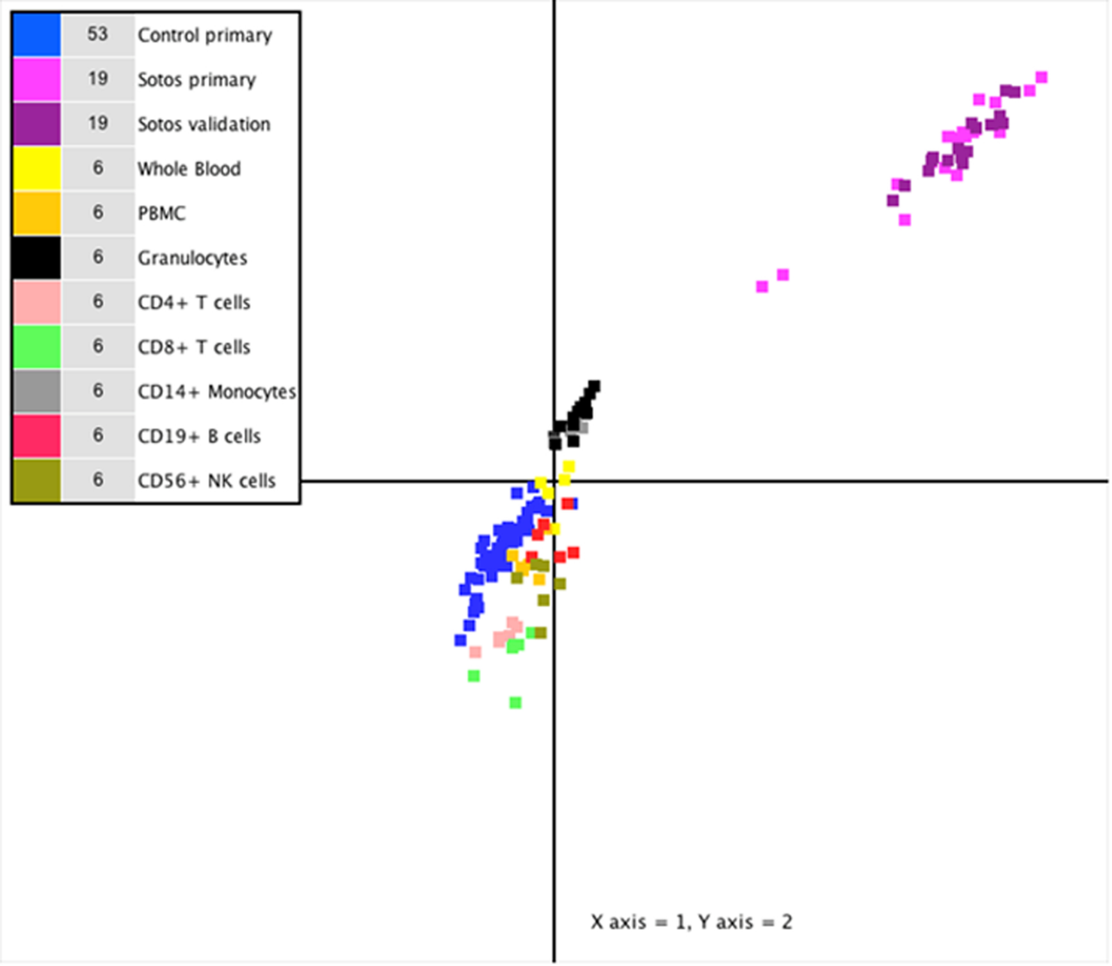

Supplementary Figure 2. Comparison of the *NSD1*$^{+/-}$ specific signature with blood cell- type composition. We extracted DNAm data from Reinius et al. (2012); GEO series GSE35069 representing 6 each of the following cell types: whole blood, peripheral blood mononuclear cells (PBMC), granulocytes as well as isolated cell populations (CD4+ T cells, CD8+ T cells, CD56+ NK cells, CD19+ B cells, CD14+ monocytes). Using the *NSD1*$^{+/-}$ specific signature, we plotted using Principal Component Analysis the 48 blood cell-type samples against the 19 SS and 53 control samples from the discovery cohort. All normal blood samples, including purified blood subtypes and whole blood from the discovery cohort and from Reinius et al. (2012), are well separated from the SS samples demonstrating the robustness of the *NSD1*$^{+/-}$ specific signature.

Supplementary Methods

A. Sample Collection

1. Discovery cohort

Individuals with a clinical diagnosis of Sotos syndrome (SS) and a pathogenic *NSD1* mutation were recruited through the Division of Clinical and Metabolic Genetics at the Hospital for Sick Children in Toronto, Ontario and Our Lady's Hospital for Sick Children in Dublin, Ireland. A clinical diagnosis of Sotos syndrome was established based on the following criteria: height greater than 2 SD above the mean, macrocephaly (OFC >2SD), developmental delay and characteristic facial gestalt[1]. Informed consent was obtained from parents of all participants and assent was obtained from participants, as appropriate for age. The study was approved by the Research Ethics Board at the Hospital for Sick Children. DNA from blood samples was extracted by standard methods. In all, 19 individuals, including one familial case with three affected individuals (a father and two children), were included. Only SS patients with pathogenic *NSD1* mutations such as whole gene deletion or truncating mutations were included in the discovery cohort.

We obtained skin derived fibroblasts from three SS patients with loss of function mutations in *NSD1*. The specific *NSD1* mutations and clinical features of our discovery cohort are summarized in Supplementary Table 1.

In addition, six individuals with an overgrowth phenotype (some with a suspected clinical diagnosis of SS and some without the SS gestalt) who were identified to have missense mutations (single nucleotide substitutions) classified as variants of unknown significance (VOUS) were also enrolled in the study. All patients with missense mutations were examined in person, or via medical records and photographs, independently by two of the authors with extensive clinical experience with SS (RW and DC). At the time of these assessments, RW and DC were blinded to the DNA methylation (DNAm) results.

1. Validation cohort

An additional 19 patients with a clinical diagnosis of Sotos syndrome and confirmed pathogenic *NSD1* mutations (whole gene deletion or truncating mutations) (Supplementary Table 5) and 10 patients with missense mutations were obtained from the University of Hong Kong (Supplementary Table 7).

2. Control cohorts

Genomic DNA derived from blood samples of 53 control and 4 fibroblast samples from 4 controls were used. All 53 control subjects were recruited at The Hospital for Sick Children (for detailed information about these controls, see Supplementary Table 2). To carry out stringent specificity analysis using an independent set of controls, DNAm data from an additional 1056 control blood samples were downloaded from the GEO public database (http://www.ncbi.nlm.nih.gov/geo/). These controls were deliberately selected to encompass a range of ages, both sexes and multiple batches. As well, the genomic DNA had been extracted using different methods in multiple laboratories around the world. The purpose of this selection was to test the predictive value of the $NSD1^{+/-}$ specific signature as a novel functional diagnostic tool for Sotos syndrome in the context of the commonly found biological and technical variations in such datasets.

B. Analysis of Confounding Factors

1. Blood cell-type proportions

To assess if the consensus $NSD1^{+/-}$ specific signature is affected by differences in cell proportions, we compared our data to 6 controls with 8 sorted blood cell types each, [2]which are available from GEO (series GSE35069). These data represented DNAm of the following cell types: whole blood, peripheral blood mononuclear cells (PBMC), granulocytes as well as isolated cell populations (CD4+ T cells, CD8+ T cells, CD56+ NK cells, CD19+ B cells, CD14+ monocytes). We extracted the DNAm values corresponding to the *NSD1* classification signature CpG sites (7,085 CpGs) for the 48 different blood subtype samples as well as for the 19 SS and

53 control samples used to generate the *NSD1*$^{+/-}$ specific signature. Principal component analysis was then applied to the resulting collection of 120 samples in order to detect patterns of similarity across the various data subgroups.

2. Effects of sex, age and batch

The identified *NSD1*$^{+/-}$ specific signature comprising 7,085 CpG sites was examined for the influence of confounding factors using regression analysis. Following a process similar to the one described above, we formed three separate testing trials, one for each of the familial SS patients. We then compared the DNAm distributions in SS patients against controls at every CpG site using a linear regression model in which the DNAm level was the dependent variable, the disease status (SS or control) was the independent variable, and sex, age and batch were fixed effects (implemented in R; scripts available upon request. Note that the SS and control samples from the discovery cohort were distributed over 4 different batches). Guided by the initial signature derivation, we defined two criteria for the signature CpGs: (a) the magnitude of the regression coefficient corresponding to the disease component, which indicates the average DNAm difference due to SS status, should exceed 20%; (b) its p-value should satisfy the significance level $p<0.05$ after a stringent Bonferroni correction (based on the initial 424,586 CpGs in the methylation array). P-value after regression analysis and delta beta effects for at least one of the 3 familial trials were added to Supplementary Table 3 for each CpG sites in the *NSD1*$^{+/-}$ specific signature.

Supplementary References

1. Tatton-Brown, K. & Rahman, N. Sotos syndrome. *Eur J Hum Genet* **15**, 264-71 (2007).

2. Reinius, L.E. *et al.* Differential DNA methylation in purified human blood cells: implications for cell lineage and studies on disease susceptibility. *PLoS One* **7**, e41361 (2012).
